# TOP2A as marker of response to pegylated lyposomal doxorubicin (PLD) in epithelial ovarian cancers

**DOI:** 10.1186/s13048-019-0492-6

**Published:** 2019-02-13

**Authors:** Eleonora Ghisoni, Furio Maggiorotto, Fulvio Borella, Gloria Mittica, Sofia Genta, Gaia Giannone, Dionyssios Katsaros, Alberto Sciarrillo, Annamaria Ferrero, Ivana Sarotto, Jessica Erriquez, Maria Flavia Di Renzo, Massimo Aglietta, Giorgio Valabrega

**Affiliations:** 1 Department of Oncology, University of Torino, Torino, Italy; 2 Candiolo Cancer Institute-FPO- IRCCS, Strada Provinciale 142 km 3.95, 10060 Candiolo, Turin, Italy; 3 Department of Surgical Sciences, Gynecology, AOU Città della Salute, Torino, Italy; 4 Department of Gynecology and Obstetrics, University of Torino, Mauriziano Hospital, Torino, Italy

**Keywords:** Ovarian cancer, Topoisomerase 2 alpha, Pegylated liposomal doxorubicin

## Abstract

**Objective:**

Relapsed epithelial ovarian cancer (EOC) is frequently treated with pegylated liposomal doxorubicin (PLD). Unfortunately, most patients do not benefit from treatment. Prediction of response is crucial to optimize PLD use and avoid unnecessary toxicities. We aimed at assessing the value of topoisomerase II alpha (TOP2A) expression as predictive marker of response to PLD-based therapy in patients with relapsed EOCs.

**Methods:**

We retrospectively analyzed Formalin Fixed Paraffin Embedded (FFPE) tissues from 101 patients with platinum resistant (PR) or partially platinum-sensitive (PPS) EOCs treated with PLD-based chemotherapy beyond second line in three referral cancer centers between January 2010 and June 2018. TOP2A expression was measured by immunohistochemistry (IHC): images of each sample were acquired by optical microscope and analyzed by using automatic counter software. Correlation between TOP2A expression and response to PLD was assessed. Since no cut-off for positivity has been validated yet, we dichotomized TOP2A expression based on a cut-off of 18% (mean value in this study).

**Results:**

TOP2A expression beyond cut-off was not prognostic for primary platinum-free interval in our series (*p* = 0.77) neither for optimal cytoreduction (*p* = 0.9). TOP2A > 18% was associated with a longer time to progression (TTP) following PLD-treatment, although not statistically significant (*p* = 0.394). No difference was observed between PR and PPS patients’ groups (*p* = 0.445 and *p* = 0.185, respectively). Not unexpectedly, patients with TOP2A expression > 18% treated with PLD monotherapy achieved a longer TTP compared with PLD-doublet therapy (*p* = 0.05).

**Conclusions:**

Our data suggest that TOP2A status might predict activity of PLD in patients with PR/PPS EOCs.

## Introduction

Epithelial ovarian cancer (EOC) is the leading cause of death among women with gynecological malignancies with 22,400 estimated new cases in 2017 [1]. Optimal cytoreduction to no residual disease followed by adjuvant chemotherapy with platinum-taxane combination is the standard of care [2, 3]. However, over 70% of patients with advanced stage diseases experience relapse after front-line treatment [4]. Residual disease (R) at primary surgery and sensitivity to first-line platinum-based chemotherapy (PFI, platinum-free interval) still represent the major determinants of clinical outcome [5, 6]. For partially platinum-sensitive patients (PPS), defined as progressed between six and twelve months after conclusion of first line platinum-therapy, standard options include a second line platinum-based-doublet chemotherapy or pegylated liposomal doxorubicin [PLD] - trabectedin. For platinum resistant ovarian cancers (PR), defined as progressing within 6 months from last platinum therapy, limited and debatable options are available [7]. In these patients, subsequent single-agent chemotherapies with non-platinum drugs show limited activity [8]. The most effective drugs include paclitaxel, gemcitabine, etoposide, topotecan and PLD [9–12].

PLD is one of the preferred drugs due to its favorable toxicity profile (absence of alopecia, limited cardiac toxicity, and only moderate hematological and skin toxicity) [13, 14]. However, response rates range from 16 to 25%. Consequently, to optimize the use of PLD and avoid unnecessary toxicities, prediction of response is crucial.

*TOP2A* gene is located on the locus q21 of chromosome 17, close to the HER2 gene, and is responsible for coding the nuclear enzyme type 2 topoisomerase alpha (TOP2A) [15]. TOP2A plays a key role in DNA stability and represents one of the targets of chemotherapeutic agents, such as etoposide and anthracyclines [16–18]. Several retrospective analyses have already suggested a correlation between TOP2A status and response to anthracyclines in breast cancer, both as neoadjuvant and adjuvant treatment [19–23]. Conversely, in EOCs few studies have investigated the prognostic and predictive role of TOP2A. Heterogeneous results are mainly related with the use of different detection techniques such as immunohistochemistry (IHC), Real-Time Polymerase Chain Reaction (RT-PCR) and Fluorescent-In Situ Hybridization (FISH) [24–26].

The aim of our study was to assess the correlation between TOP2A protein expression and clinical outcome of patients following PLD-based treatment in both platinum partially-sensitive and platinum-resistant patients.

## Materials and methods

### Patients’ characteristics

We screened a total of 128 patients with PR/PPS EOCs treated with PLD-based chemotherapy beyond second line in three different referral centers (Candiolo Cancer Institute FPO/IRCCS, Ordine Mauriziano Hospital and Sant’Anna Hospital) between January 2010 and April 2018. Among these, 27 cases were excluded due to non-complete medical records or unavailability of tumor material for IHC analysis. For each of the 101 selected patients, the following clinico-histopathological data were recorded: i) age at diagnosis; ii) morphological features of ovarian cancer including tumor histotype, grade (according to World Health Organization Classification of Tumours of Female Reproductive Organs, 4th Edition) and stage (according to the International Federation of Gynecology and Obstetrics [FIGO]); iii) PFI (Platinum-Free Interval) to last platinum-based therapy; iv) number of previous lines before PLD; v) PLD-based treatment scheme; vi): Cancer Antigen 125 (CA-125) pre- and post-PLD treatment; vii) best radiological response according to RECIST 1.1 criteria, if available; viii) BRCA status; ix) date of death or last follow up (FU). Complete patients’ clinical data are reported in Table 1.

**Table 1:** Patients’ characteristics

Patient number	101
Median age (years)	60
Histological subtype	
Serous	79 (78, 3%)
Endometrioid	7 (7%)
Mucinous	2 (1, 9%)
Clear cell	2 (1, 9%)
Mixed	3 (2, 9%)
Other/not specifed	8 (8%)
Grading	
G1	0
G2	7 (7%)
G3	94 (93%)
Staging (FIGO)	
IC	1 (0, 9%)
IIA	4 (3, 9%)
IIB	7 (7%)
IIIA	4 (3, 9%)
IIIB	8 (8%)
IIIC	56 (55, 5%)
IV	21 (20, 8%)
PFI	
< 6 months	65 (64, 4%)
≥ 6 months	36 (35, 6%)
Number of previous chemoterapies	
1	82 (87, 3%)
2	10 (10, 6%)
≥ 3	2 (2, 1%)
PLD-based regimen	
PLD monotherapy	54 (53, 4%)
Carbo-PLD	30 (29, 7%)
Trabe-PLD	17 (16, 9%)
CA-125 mean value (UI/mL)	
Pre-PLD treatment	1142
Post-PLD treatment	1493
BRCA status	
BRCA 1–2 mutation	11 (10, 9%)
BRCA 1–2 wild-type	12 (11, 9%)
VUS/HRD	6 (5, 9%)
Unknown	72 (71, 3%)

*FIGO* International Federation of Gynaecology and Obstretics, *PFI* Platinum Free Interval from first platinum-based therapy, *PLD* Pegylated Liposomal Doxorubicin, *Carbo* Carboplatin, *Trabe* Trabectidin, *CA-125* Cancer Antigen 125

### IRB approval

In Italy, the National Regulation established that retrospective studies require a notification to the local ethical committee with the tacit consent formula. We therefore notified the Candiolo Cancer Institute ethical committee about the conduct of the study on August 2016. All patients included in our retrospective study were treated according with the ethical standards of our local committee on human experimentation and with the Helsinki Declaration.

### Immunoistochemistry

FFPE tissue blocks of the above 101 cases were obtained from the archives of the Units of Pathology at Candiolo Cancer Institute (IRCCS), A.S.O. Ordine Mauriziano Hospital and Sant’Anna Hospital at Torino. Immunohistochemistry was performed as previously described [27] to detect topoisomerase II alpha using a monoclonal rabbit antibody (clone D10G9 Cell Signaling Technology). At least 10 images of each sample were acquired by optical microscope (20×) connected with charge-coupled device (CCD) camera and analyzed by using automatic counter software (NIH ImageJ). Only cells with TOP2A nuclear signatures were considered as positive. TOP2A expression was calculated as the ratio between positive cells and a total of at least 500 cells in 10 different fields.

### Statistical analysis

Time to progression (TTP) following PLD-based treatment was assessed as the time elapsed between PLD start and first tumor progression or death, whichever comes first. TTP was estimated by the Kaplan-Meier method and compared according to TOP2A expression using the log-rank test. Because no positivity cut-off has been validated, similar to other studies we dichotomized TOP2A expression based on the mean value of our cohort (18%). A *p* < 0.05 was considered statistically significant. All analyses were performed using the SPSS statistical software program, version 22.0 (IBM SPSS Inc., Chicago, IL, United States of America).

## Results

In our series, more than 75% were serous high grade, G3, advanced stage (IIIC-IV) EOCs. PFI showed that almost 65% of the patients were platinum-resistant. Eighty-seven per cent of the patients received PLD as second-line therapy and more than 50% as single-agent.

**Fig. 1:**
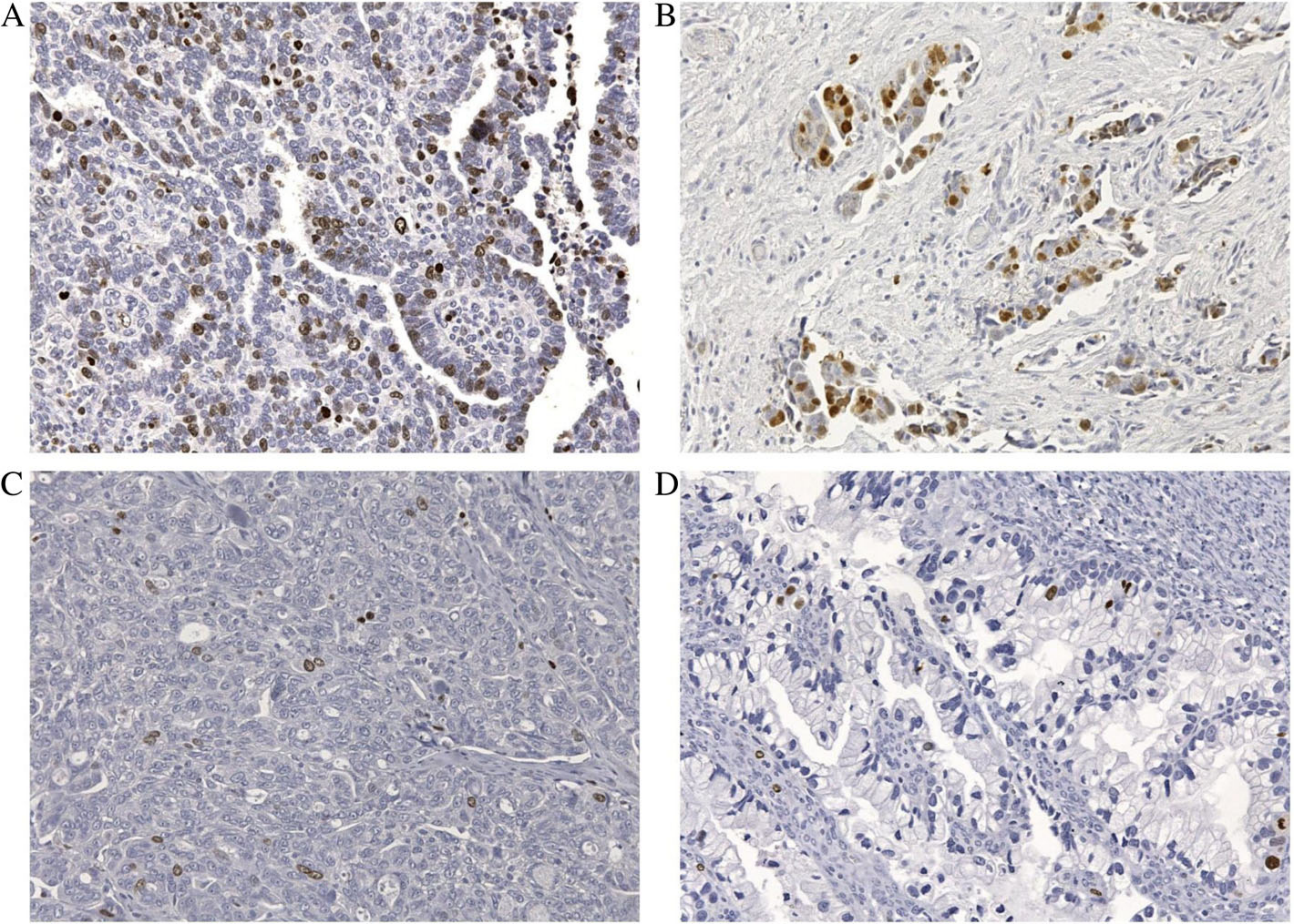
TOP2A expression detected at IHC (images acquired by optical microscope 20×). **a**, **b** TOP2A over-expression above cut-off of 18% nuclear cells signature versus. **c, d** TOP2A expression below cut-off

All 101 cases analyzed showed nuclear TOP2A expression, which ranged from 2 to 48% of nuclear cell signature with a mean of 18% (Fig. 1).

No statistically significant association between TOP2A expression and tumor histotype, grading or PFI were found. TOP2A expression beyond cut-off was not prognostic for primary platinum-free interval in our series (*p* = 0.77) neither for optimal cytoreduction (*p* = 0.9).

**Fig. 2:**
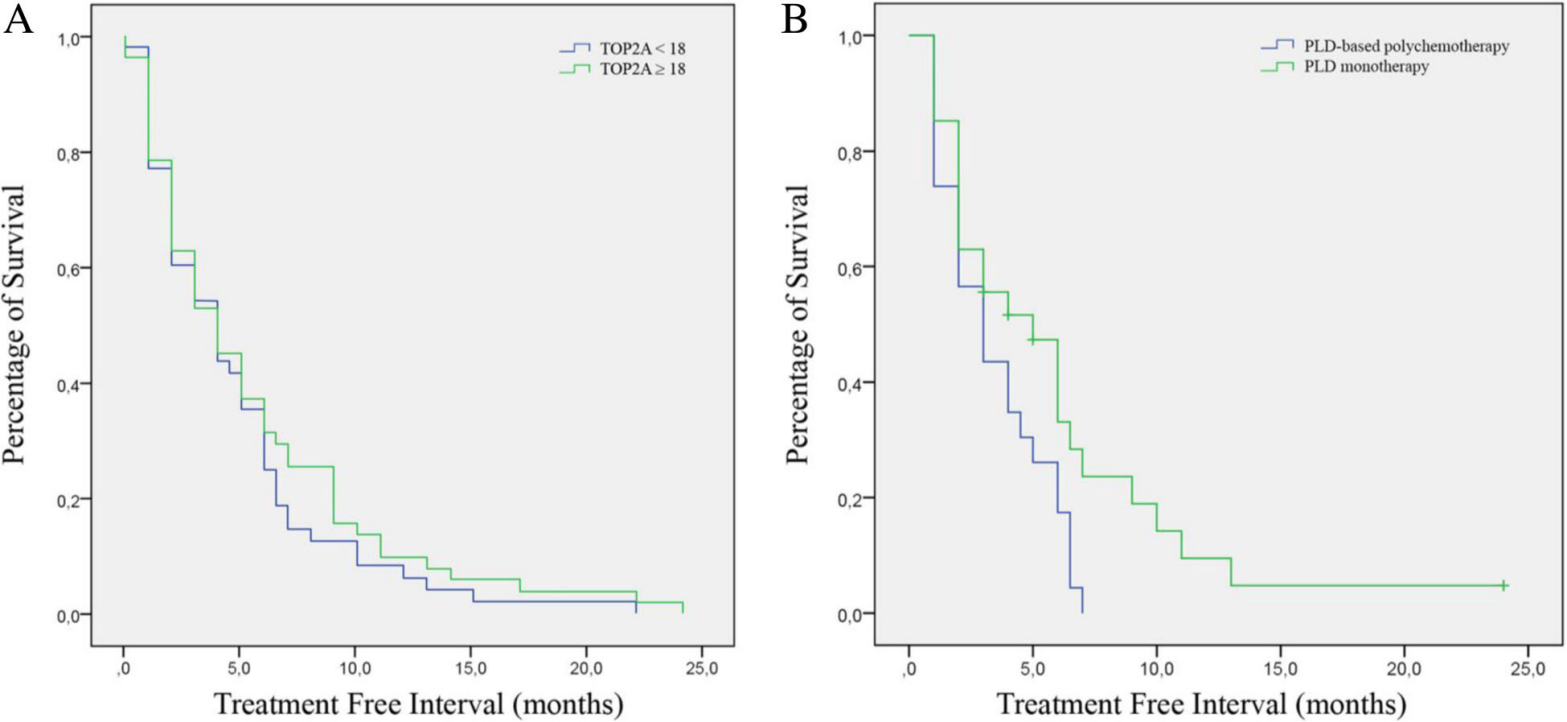
Correlation between TOP2A expression and PLD-Treatment Free Interval. **a** Cases showing TOP2A expression above 18% (mean value of this study) versus cases with TOP2A below cut-off limit. **b** Patients with TOP2A expression above 18% treated with PLD-monotherapy versus PLD-based polychemotherapy

Median time to progression from PLD-based treatment was 5, 1 months (range 1–24 months). Cancer Antigen 125 (CA-125) was above normal range in the 93% of cases before PLD treatment (median value 1142 UI/dL) and in the 78, 3% post-treatment (median value 1493 UI/dL), but not statistical correlation was found between serological response and TOP2A status (*p* = 0.285). TOP2A expression beyond cut-off was associated with a higher probability of response to PLD in terms of TTP, although not statistically significant (*p* = 0.0394, Fig. 2a). No difference was observed between PR and PPS groups (*p* = 0.445 and *p* = 0.185, respectively). Among the 67 patients with available radiological assessments, no difference could be observed in term of response: 31 patients obtained a disease control (partial response [PR] + stable disease [SD]) and 36 progressed. Patients with TOP2A higher expression treated with PLD monotherapy achieved a longer TTP compared with PLD-doublet therapy (5, 8 vs 3, 4 months, *p* = 0.035, Fig. 2b). Complete results are summarized in Table 2.

**Table 2:** Correlation between TOP2A expression and response to Pegylated Lyposomal Doxorubicin (PLD) in terms of time to progression (TTP)

Time To Progression (TTP) to PLD	TOP2A < 18%	TOP2A ≥ 18%	*p*-value
All patients, months (CI 95%)	4,5 (3,1-5,9)	6,7 (4,8-8,6)	0,085
TTP according to Platinum Free Interval			
PR	3,7 (2,6-4,9)	4,9 (2,9-6,9)	0,445
PPS	5,6 (2,7-8,6)	9,1 (5,8-12,4)	0,185
TTP according to chemoterapeutic regimen			
PLD monotherapy	3,4 (2,5-4,3)	5,8 (3,7-3,9)	0,035
Carboplatin-PLD	8,0 (3,5-12,4)	9,6 (5,7-13,5)	0,796
PLD-Trabectidin	1,8 (0,6-3,2)	3,5 (0,6-6,3)	0,358

*TTP* time to progression, *CI* confidence interval, *PR* Platinum-resistant patients, *PPS* partially platinum-sensitive patients

BRCA germline-status was known in 29 patients. Among the eleven mutated patients, 7 carried a BRCA1 and 4 a BRCA2 mutations; twelve patients were wild-type and other six patients showed a variant of unknown significance (VUS). As expected, patients with BRCA mutations achieved a longer TTP (median 11 months, range 3–22, data not shown) compared with the median of our study population, although statistical analysis could not be performed due to the small numbers of cases.

## Discussion

Treatment of patients affected by relapsed PR or PPS EOCs is still a major challenge for gynecologic oncologists and medical oncologists. To personalize the use of PLD and avoid unnecessary toxicities, prediction of response is of crucial importance. We have already demonstrated that *TOP2A* gene copy number is associated with protein overexpression and correlates with the activity of PLD in a small series of 38 PR EOCs and patients-derived xenografts (PDXs) [28]. Considering the concordance between *TOP2A* gene copy number and TOP2A protein expression, we aimed at developing a cost-effective and reproducible method (IHC) to assess the correlation between TOP2A and PLD activity. In our study we found that the prevalence of TOP2A expression is consistent with that observed by van der Zee at al [29]. and Faggad et al. [26] in their retrospective series. Moreover, we report here that TOP2A expression above 18% is associated with a higher probability of response to PLD in patients with PR/PPS EOCs. Finally, we showed that, regardless platinum-free interval, patients with TOP2A expression above cut-off, achieved a longer time to progression if treated with PLD monotherapy compared with PLD-doublet therapy (*p* = 0.035). These data may be explained by the known detrimental effect of poly-chemotherapy in pretreated patients [30, 31].

To our knowledge this is the biggest study investigating the role of TOP2A in predicting PLD activity in ovarian cancers.

However, our study has several limits. First of all the absence of a validated cut-off for TOP2A positivity at IHC impairs the reproducibility of the test. Moreover, assessment of TOP2A expression mainly at diagnosis may be discordant with TOP2A status at relapse, considering tumor heterogeneity and clonal evolution during progression. Finally our patient population was heterogeneous in terms of PFIs and PLD combinations.

Other predictors of sensitivity to anthracyclines have been studied. Among these, chromosome 17 polysomy, HER2, TIMP-1 [32], stroma-related genes and the immune response [33] have been already investigated with controversial results. More recently, defects in the homologous recombination system, such as BRCA1 and BRCA2 mutations have been associated with higher rates of response to platinum-based and anthracyclines regimens both in triple negative breast cancers (TNBCs) and EOCs [34].

In conclusion, although we believe that these results should be regarded as an hypothesis-generating attempt to identify a biomarker of response, only a multifactorial panel could be predictive of response to PLD in ovarian cancer [35].

## Abbreviations

CA-125: Cancer Antigen 125
Carbo: Carboplatin
ECOG: Eastern Cooperative Oncology Group (ECOG)
EOC: Epithelial ovarian cancer
FFPE: Formalin Fixed Paraffin Embedded
FIGO: International Federation of Gynecology and Obstetric
FISH: Fluorescent-In Situ Hybridization
IHC: Immunohistochemistry
PFI: Platinum-free interval
PLD: Pegylated liposomal doxorubicin
PPS: Partially platinum-sensitive
PR: Platinum-resistant
PS: Performance status
R: Residual tumor
RT-PCR: Real-Time Polymerase Chain Reaction
TNBCs: Triple negative breast cancers
TOP2A: Topoisomerase II alpha
Trabe: Trabectedin
TTP: Time to progression
VUS: Variant of unknown significance
